# Do inhaled or oral glucocorticoids more effectively control feline asthma?

**DOI:** 10.18849/ve.v7i4.560

**Published:** 2022-12-07

**Authors:** Savannah Williams+

**Keywords:** FELINE THERAPY, GLUCOCORTICOIDS, INHALED THERAPY, ORAL THERAPY, TREATMENT

## Abstract

PICO question

In cats with chronic bronchospasm and airway hypersensitivity (asthma) do oral glucocorticoids or inhaled glucocorticoids more effectively control the clinical signs?

Clinical bottom line

Category of research question

Treatment.

The number and type of study designs reviewed

Three prospective randomised clinical trials were appraised. Two of the studies followed a crossover design and had a control group, whilst the third study described an interrupted time series.

Strength of evidence

Weak.

Outcomes reported

The available studies deemed a reduction in eosinophilia on bronchoalveolar lavage fluid analysis, and a reduction in airway resistance as markers of treatment efficacy.

Conclusion

There is weak evidence to suggest equal treatment efficacy of oral and inhaled glucocorticoid therapy for management of feline asthma. Higher powered studies would be required before a definitive recommendation can be made.

How to apply this evidence in practice

The application of evidence into practice should take into account multiple factors, not limited to: individual clinical expertise, patient’s circumstances and owners’ values, country, location or clinic where you work, the individual case in front of you, the availability of therapies and resources.

Knowledge Summaries are a resource to help reinforce or inform decision making. They do not override the responsibility or judgement of the practitioner to do what is best for the animal in their care.

## Clinical scenario

You are presented with a 4 year old cat with a 5 month history of intermittent coughing alongside periods of acute wheezing and respiratory difficulty. Clinical examination of the cat is unremarkable. Investigations inclusive of bloodwork, thoracic radiography and a bronchoalveolar lavage reveal an eosinophilia, bronchial pattern, and eosinophilic infiltrate, respectively. A faecal Baermann test was negative for lungworm larvae. A diagnosis of feline asthma is made. You wish to start the cat on glucocorticoids however, you are unsure whether oral therapy or inhalant therapy is more effective at controlling clinical signs.

## The evidence

Database searches identified three papers offering evidence to answer the PICO question (Verschoor-Kirss et al., 2021; Leemans et al., 2012; and Reinero et al., 2005) once duplicates had been removed. All studies were prospective in nature with two of them following a crossover design (Leemans et al., 2012; and Reinero et al., 2005). None of the studies were directly comparable due to differences in study population type and differing drug regimens, although the outcomes measured were similar.

## Summary of the evidence

**Verschoor-Kirss et al. (2021):** 

Population:	Naturally asthmatic cats that had not received treatment previously.
Sample size:	Nine cats.
Intervention details:	Cats were randomised into two study groups: Four cats received 5 mg oral prednisolone BID for 2 weeks, then SID for 6 weeks; Five cats received 5 mg oral prednisolone SID and110 ug of inhaled fluticasone BID for 1 week then 110ug of inhaled fluticasone BID exclusively for 7 weeks.
Study design:	Prospective non-blinded randomised clinical pilot trial.
Outcome Studied:	Changes on thoracic radiography. Lung function testing (airway resistance). Total nucleated cell count and percentage of eosinophil count from blind bronchoalveolar lavage (BAL) samples. Serum fructosamine and serum allergy testing were assessed before (baseline) and after (8 weeks post) treatment.
Main Findings (relevant to PICO question):	After 8 weeks of treatment both groups were absent of clinical signs of asthma as perceived by their owners and clinicians. There was no significant difference between pre and post-treatment thoracic radiographs, as assessed by a semi-quantitative scoring system. Airway eosinophilia decreased significantly in all cats (P = 0.003) regardless of treatment modality. 3/4 (75%) cats (on oral therapy reached the therapeutic target point for airway eosinophilia compared to 2/5 (40%) cats treated with inhalant therapy. Improvement in airway resistance was more notable in cats treated with oral glucocorticoids although baseline resistance was higher in this group. There was no relationship (r = 0.27) between the percentage of eosinophils in BAL fluid and airway resistance. Despite therapy, airway eosinophilia and increased airway resistance remained in several cats, even though they appeared clinically normal. Both oral and inhalant glucocorticoid therapy improved lung function and airway eosinophilia.
Limitations:	Small study population. Underpowered study. No placebo group. The cat population underwent lung function testing having not been aerosol challenged, therefore it may not be representative of an asthmatic cat.

**Leemans et al. (2012):** 

Population:	Cats with experimentally induced asthma sensitised to *Ascaris suum *allergen.
Sample size:	Six cats.
Intervention details:	Feline asthma was induced via two intramuscular injections of *Ascaris suum *allergen, 2 weeks apart, and again 2–4 weeks later followed by a 5 minute long inhalation challenge with 0.01% aerosolised *Ascaris suum *allergen. All cats received each treatment modality with a 4 week recovery interval between each round of treatment. Then in a crossover design, the following treatments were administered for 4 days: Oral prednisolone (1 mg/kg BID). Fluticasone propionate (500 ug BID) via a metered dose inhaler. Combination of inhaled fluticasone propionate and salmeterol (500 ug/50 ug) BID. Untreated. On day 2 of the treatment course each cat underwent a single 5 minute challenge with 0.01% aerosolised Ascaris suum allergen.
Study design:	Prospective randomised crossover clinical trial.
Outcome Studied:	Thoracic radiological assessment via a semi-quantitative scoring system. Lung function and airway responsiveness (by barometric whole-body plethysmography [BWBP]). This was assessed immediately prior to airway sensitisation and then at: 5 min, 15 min, 1 hour and 2 hours after challenge, and subsequently every 2h until 8h post challenge. Haematology and matrix metalloproteinase (MMP) analysis. Bronchoscopic findings. Bronchoalveolar lavage (BAL) fluid assessment: differential cell counts including percentage eosinophils, total protein content, 8-iso-PGF2a concentration, MMP analysis.
Main Findings (relevant to PICO question):	There was no significant difference in BWBP between treatment groups. All medications caused a reduction in BAL fluid eosinophil percentage, with the oral and combined treatments causing a significant reduction. Fluticasone given as monotherapy was less effective than oral prednisolone at reducing the severity of airway eosinophilia. Radiological score and bronchoscopic score did not differ between treatment groups (data not shown).
Limitations:	Small cohort. Lack of crossover evaluation of untreated cats. Experimentally induced asthmatic cats may not actively reflect the changes of natural asthmatic cats. Cats were not sedated for thoracic radiography and only ventrodorsal and right lateral views were taken which may have affected scoring.

**Reinero et al. (2005):** 

Population:	Cats sensitised to Bermuda grass allergen (BGA).
Sample size:	Six cats.
Intervention details:	Feline asthma was induced via subcutaneous administration of BGA and sensitisation was confirmed via intradermal skin testing and aerosol challenge.Cats were then exposed to each treatment for 2 weeks: Prednisone 5 mg PO q 12h. Flunisolide 250 ug inhaled using a metered dose inhaler q 12h. LT-receptor antagonist (Zafirlukast) 10 mg PO q 12h. Antiserotonergic (Cyproheptadine) 2 mg PO q 12h. Control substance. A 4 week wash out period was maintained in between each treatment and subsequent challenge.
Study design:	Prospective randomised placebo controlled crossover.
Outcome Studied:	Airway resistance. Percentage of eosinophils in BAL fluid. Blood lymphocyte phenotype. Serum content of allergen specific immunoglobulin E (IgE). Serum and BAL fluid content of allergen specific immunoglobulin G (IgG) and immunoglobulin A (IgA).
Main Findings (relevant to PICO question):	Airway resistance was not consistently reduced by any of the drugs in the study. Cats that received oral prednisolone or inhaled flunisolide treatments had a significantly reduced mean percentage of eosinophils detected in BALF compared to control. Inhaled flunisonide did not significantly decrease airway hyperresponsiveness in all cats.
Limitations:	Small cohort. Disease model may not reflect all facets of natural asthma.

## Appraisal, application and reflection

All three studies used a reduction in airway resistance and reduction in airway eosinophilia as measures of treatment success (Verschoor-Kirss et al. 2021; Leemans et al. 2012; and Reinero et al. 2005).

Pulmonary function testing of asthmatic cats offers a non-invasive means of assessing response to therapy, and in the study by (Leemans et al. 2012) barometric whole-body plethysmography was used in unrestrained conscious cats to assess changes in airway resistance. In contrast, Reinero et al. (2005) and Verschoor-Kirss (2021), anaesthetised cats before airway challenge commenced and the airway’s response was assessed. Leemans et al. (2012) and Reinero et al. (2005) did not find a significant difference in the reduction of airway resistance among treatment groups. Meanwhile Verschoor-Kirss (2021) found that there was a greater reduction in airway resistance with oral glucocorticoid treatment compared to baseline than with inhaled therapy. Although this study does highlight that cats in the inhaled treatment group started with higher baseline resistance than those in the oral group.

Regardless of the study design all studies found that when an oral or inhaled therapy was used, treatment resulted in a reduction in airway eosinophilia. However, only Leemans et al. (2012) found the decrease in BAL fluid eosinophil percentage to be more significant with oral therapy compared to inhaled therapy alone. Interestingly, when salbutamol was used concurrently with the inhaled therapy in this study, they found a significant decrease in BAL fluid eosinophil percentage also (Leemans et al. 2012).

An additional method that was used to evaluate treatment response in the asthmatic cat cohorts, was the change in radiographic appearance of the lungs (determined by a clinical score). Verschoor-Kirss (2021) and Leemans et al. (2012) used changes in the radiographic appearance of the lung as a study outcome. Thoracic radiographs were assessed before and after treatment administration and assigned a score. However, no significant difference was detected between scores before or after oral or inhaled treatment in either study.

Two of the three papers chose to assess fluticasone propionate as the inhaled glucocorticoid and prednisolone as the oral glucocorticoid (Verschoor-Kirss et al. 2021; and Leemans et al. 2012) whilst Reinero et al. (2005) assessed flunisolide and prednisone. Despite two of the studies using the same medications, they were not cross comparable as the doses of the drugs, and length of time for which cats received the oral and / or inhaled glucocorticoid treatment varied greatly. Treatment duration varied from 4 days (Leemans et al. 2012) to 8 weeks (Verschoor-Kirss et al. 2021). Future studies could consider using varying concentrations of each treatment on the same study population to determine the minimum effective dose to control clinical signs associated with asthma.

Verschoor-Kirss et al. (2021) was the only study that assessed a population of naturally asthmatic cats. Clinically their response to treatment is more likely to reflect that of owned asthmatic cats. The treatment protocol in this study differed from the other studies; oral glucocorticoid treatment was given concurrently with the inhalant therapy for 1 week before inhalant therapy was continued exclusively. Whilst this makes it more difficult to determine the efficacy of fluticasone as a sole therapy, this treatment protocol is more likely to be reflective of a feline asthma treatment protocol prescribed in general practice.

All the clinical studies had low numbers of feline participants and study duration was short. The small cohort numbers reduce the statistical power of each paper’s results and consequently make it difficult to draw confident conclusions. Power analysis would be beneficial before performing further prospective, randomised, blinded studies.

Overall, there is a weak pool of evidence available to determine if oral or inhaled glucocorticoid treatment is more effective at managing the airway inflammation associated with feline asthma.

## Methodology Section

**Search Strategy:** 

Databases searched and dates covered:	CAB Abstracts 1973 to 2021 Week 46 (via the OVID platform)Ovid MEDLINE® 1946 to present (via the OVID platform)
Search strategy:	The same search was used for both databases: (cat or cats or feline*).mp. or exp cats/ (asthma* or wheez* or respiratory hypersensitiv* or airway hyper responsiveness or airway hyper-responsiveness or bronchospasm* or bronchial spasm*).mp. or exp asthma/ (glucocorticoid* or glucocorticosteroid*).mp. or exp glucocorticoids/ 1 and 2 and 3
Dates searches performed:	10 Apr 2022

**Exclusion / Inclusion Criteria:** 

Exclusion:	Articles where the full text was not available in English; articles not relevant to the PICO question; articles listed as: a single case report, conference proceedings, book chapter, or review article.
Inclusion:	Articles written in English; relevant to PICO question; more than one animal; studies that used an inhaled and oral glucocorticoid as the only treatment.

**Search Outcome:** 

Database	Number of results	Excluded – Not relevant to the PICO question	Excluded – Not available in English	Excluded – Book chapter / conference proceeding / single case report / review article	Total relevant papers
CAB Abstracts	33	17	5	10	1
OVID Medline®	55	50	1	1	3
Total relevant papers when duplicates removed					3

## ORCID

Savannah Clare Williams: https://orcid.org/0000-0001-8341-9074

## Conflict of Interest

The author declares no conflicts of interest.
